# Interpretation of Non-Clinical Data for Prediction of Human Pharmacokinetic Parameters: In Vitro-In Vivo Extrapolation and Allometric Scaling

**DOI:** 10.3390/pharmaceutics11040168

**Published:** 2019-04-05

**Authors:** Go-Wun Choi, Yong-Bok Lee, Hea-Young Cho

**Affiliations:** 1 College of Pharmacy, CHA University, 335 Pangyo-ro, Bundang-gu, Seongnam-si, Gyeonggi-do 13488, Korea; gwchoi153@gmail.com; 2 College of Pharmacy, Chonnam National University, 77 Yongbong-ro, Buk-Gu, Gwangju 61186, Korea; leeyb@chonnam.ac.kr

**Keywords:** pharmacokinetics, in vitro-in vivo extrapolation, allometric scaling, animal scale-up, translational approach, non-clinical study

## Abstract

Extrapolation of pharmacokinetic (PK) parameters from in vitro or in vivo animal to human is one of the main tasks in the drug development process. Translational approaches provide evidence for go or no-go decision-making during drug discovery and the development process, and the prediction of human PKs prior to the first-in-human clinical trials. In vitro-in vivo extrapolation and allometric scaling are the choice of method for projection to human situations. Although these methods are useful tools for the estimation of PK parameters, it is a challenge to apply these methods since underlying biochemical, mathematical, physiological, and background knowledge of PKs are required. In addition, it is difficult to select an appropriate methodology depending on the data available. Therefore, this review covers the principles of PK parameters pertaining to the clearance, volume of distribution, elimination half-life, absorption rate constant, and prediction method from the original idea to recently developed models in order to introduce optimal models for the prediction of PK parameters.

## 1. Introduction

One of the main reasons associated with the termination of drug development is inappropriate pharmacokinetic (PK) properties in humans [1]. Drugability is mainly dependent on the drug’s metabolism and pharmacokinetic (DMPK) properties, which are the main hurdles in pharmaceutical R&D. Approximately 40% of drug failures are attributed to DMPK issues [2]. The main cause of failure in clinical trials is poor efficacy [3]. Although issues related to PK and bioavailability have improved since the 2000s [4], nearly half of all the therapeutic candidates in drug development are lost due to poor absorption, distribution, metabolism, excretion (ADME), toxicology, and pharmacology (safety) [5].

Therefore, the prediction of PK properties in humans before the first-in-human clinical trials is one of the main purposes of non-clinical studies in the drug discovery–development process. The two ways of predicting PK in humans include in vitro-in vivo extrapolation (IVIVE) and allometric scaling (AS).

The physiologically-based IVIVE model is based on physiological, biochemical, and biopharmaceutical factors such as organ size, blood flow rate, enzyme kinetics, drug permeability, partitioning factor into the organ, and various in vitro clearance data. These data are incorporated into the IVIVE model to provide valuable insight into drug properties and evidence to guide decision-making in the drug discovery-development process. Despite its advantages, construction of the IVIVE model requires knowledge of PKs and an understanding of complex mathematical equations. Moreover, this approach is expensive and time-consuming [6]. Although to project in vitro to in vivo data is difficult due to the complexity of the interdependent biological processes and their dynamic nature [7], it is more physiologically relevant than AS, considering that IVIVE incorporates physiological factors and includes the possibility to expand with the mechanistic model [8,9]. In IVIVE, although methods to predict the various forms of clearance are available (e.g., biliary [10], renal [11,12,13], glucuronidation [11], and hydrolysis [14] clearance), we focus on the prediction of hepatic clearance which is the primary elimination pathway. AS is an empirical approach to predict human PK parameters. The origin idea and application of AS in PKs have been discussed in detail by numerous works [15,16,17,18,19,20,21]. Although AS is empirical and has limitations for drugs with high protein bound, extensive active renal secretion, and other transport processes or have species-specific binding or distribution, that may poorly predict human PK parameters [22], it is simple and less complicated, while providing a valuable insight as well.

Although basic principles and methodologies of the two methods vary, they have a common goal which is human PK prediction. Data required for IVIVE and AS were obtained from non-clinical studies prior to the entry of clinical trials [23,24,25]. Animal PK data are routinely obtained in non-clinical drug development processes [26]. These two methods are practically used to estimate the first-in-human dose in clinical trials [25].

Until now, numerous IVIVE and/or AS methods have been developed and comparative analyses have been conducted. However, a general overview of the fundamental principle of PK parameters for the application of IVIVE and AS is lacking, and the available methods are scattered. Therefore, this review will provide a comprehensive overview of the underlying principles of PK parameters with mathematical equations.

## 2. Theoretical Background for the Prediction of Clearance

### 2.1. Physiological Clearance Concept

Clearance (CL) is considered the most important PK parameter as it is related to drug elimination and bioavailability [27]. Further, the main purpose of IVIVE is to predict human CL using in vitro data and physiologically relevant mathematical equations. Therefore, an understanding of the basic principles of the CL concept is the first step prior to applying IVIVE. Due to the significance of the parameter itself, and physiological relevance, prediction of CL is one of the key steps in drug discovery and development.

There are three methods for the calculation of CL in PKs [28].

1. Non-compartmental analysis (NCA): This method employs data-dependent and model-independent calculations without the need to define a specific compartment model. The elimination constant is derived from the linear-regression of the elimination phase of a drug. The CL in NCA is calculated using Equation (1), in which the dose is the amount of drug introduced to systemic circulation and AUC_0-inf_ is area under the concentration-time curve from zero to infinity. In this equation, the volume of distribution (V) does not need to be defined. In case of administration involving the absorption pathway, the dose is adjusted based on bioavailability (F).

(1) $$\mathrm{CL}=\frac{\mathrm{Dose}\cdot F}{{\mathrm{AUC}}_{\inf}}$$

2. Compartmental analysis: CL is calculated using the elimination rate constant (e.g., expressed as k, k_el_, or k_10_) and V. This method assumes a defined compartment model. In this method, CL is calculated based on the following equation:

(2) CL=k·V

3. Physiological model: This model describes CL by incorporating physiological, anatomical, and/or biochemical aspects. The knowledge of the physiological PK model is crucial to understand IVIVE since it has improved by the efforts to explain the PK phenomenon as more physiologically relevant. Therefore, comprehensive physiological CL concepts are described in the following subsections.

#### 2.1.1. Organ Clearance

The concept of organ clearance is based on the loss of a parent drug across an organ of elimination [29,30,31,32]. A well-perfused clearing organ exhibits the ability to clear xenobiotics. If a drug is cleared in the clearing organ, then C_out_ is less than C_in_ (C_out_ < C_in_), in which C_in_ and C_out_ indicate drug concentration in artery and venous, respectively.

The rate of the input and output of a drug can be expressed by multiplying drug concentration with flow, Q. Regarding mass balance, the rate of elimination is defined by the difference between input and output as described below.

Rate of elimination = C_in_·Q − C_out_·Q = Q·(C_in_ − C_out_) (3)

Organ extraction ratio (ER) is the ratio of the elimination rate to the input rate. Thus, ER can be understood as the efficiency with which the organ clears the drug under a specific blood flow, Q. ER is calculated using the following equation:

(4) $$\mathrm{ER}=\frac{Q\left(C_{\mathrm{in}}-C_{\mathrm{out}}\right)}{Q\cdot C_{\mathrm{in}}}=\frac{C_{\mathrm{in}}-C_{\mathrm{out}}}{C_{\mathrm{in}}}=1-\frac{C_{\mathrm{out}}}{C_{\mathrm{in}}}$$

Organ clearance is the volume of blood cleared of a drug by an organ per unit of time. It is expressed by the following equation [33]:

(5) $$\mathrm{CL}=\frac{\mathrm{The}\;\mathrm{rate}\;\mathrm{of}\;\mathrm{elimination}}{C_{\mathrm{in}}}=\frac{Q\left(C_{\mathrm{in}}-C_{\mathrm{out}}\right)}{C_{\mathrm{in}}}=Q\cdot \mathrm{ER}$$

The ER is a dimension-less parameter. As shown in Equation (5), it is obvious that the organ clearance is limited by the blood flow in the specific organ. Since ER is greater than or equal to 0 but less than or equal to 1 (0 ≤ ER ≤ 1), CL is greater than or equal to 0 but less than or equal to Q (0 ≤ CL ≤ Q).

**Figure 1:**
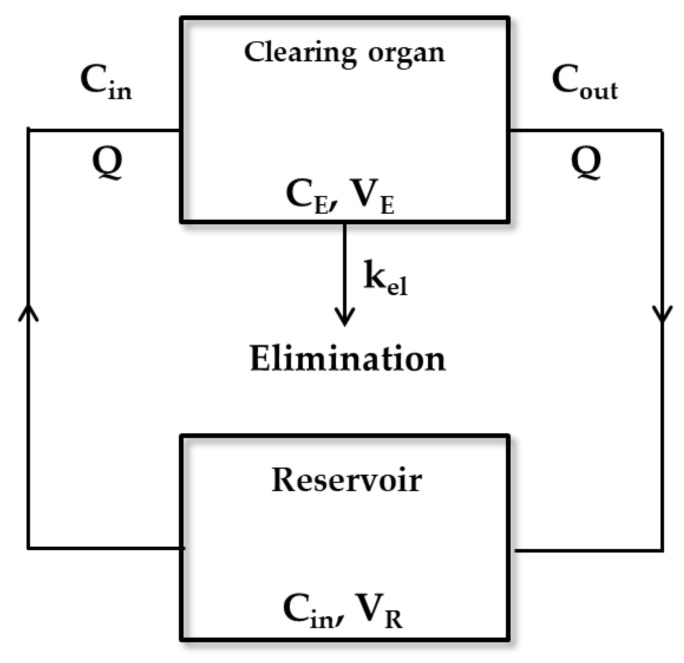
The perfusion model including one reservoir and one clearing organ. In this model, Q refers to the rate of perfusate or blood flow. C_in_ is the drug concentration in the artery entering the reservoir and clearing organ. C_out_ denotes the drug concentration in veins leaving the clearing organ and entering the reservoir, which is a non-clearing organ. V_E_ and V_R_ indicate the volume of clearing organ and reservoir, respectively. The elimination process is followed by first-order kinetics and its elimination constant is represented by k_el_. C_E_ is the drug concentration in the clearing organ.

In Figure 1, the perfusion model explains the relationship between Q and ER [34]. This model follows the well-stirred model that will be mentioned later. It assumes that the drug distribution in intra-cellular and extra-cellular fluids can instantaneously reach equilibrium, where the blood flow rate is rapid enough not to limit the distribution of a drug. If a drug is bolus administered into the reservoir, the mass balance equations are described by the following Equations (6) to (9).

Differential equations for the reservoir and clearing organs are as follows:

(6) $$-V_{R}\frac{{\mathrm{dC}}_{\mathrm{in}}}{\mathrm{dt}}=Q\left(C_{\mathrm{in}}-C_{\mathrm{out}}\right)$$

(7) $$V_{E}\frac{{\mathrm{dC}}_{E}}{\mathrm{dt}}=Q\left(C_{\mathrm{in}}-C_{\mathrm{out}}\right)-k_{\mathrm{el}}\cdot V_{E}\cdot C_{E}$$

where C_E_ is drug concentration in the clearing organ. However, in a practical setting, the analysis of the actual drug concentration in the organ is impossible. Therefore, C_E_ is substituted by C_out_ which can be measured in a practice setting using the partition coefficient between C_E_ and C_out_ as shown in the equations below:

(8) $$K_{P}=\frac{C_{E}}{C_{\mathrm{out}}}$$

(9) $$K_{P}\cdot V_{E}\cdot \frac{{\mathrm{dC}}_{\mathrm{out}}}{\mathrm{dt}}=Q\left(C_{\mathrm{in}}-C_{\mathrm{out}}\right)-k_{\mathrm{el}}\cdot K_{P}\cdot V_{E}\cdot C_{\mathrm{out}}$$

Solving Equations (7) and (9) for C_in_ and C_out_ and substituting these solutions into Equation (1), the final solution yields Equation (10) below. The detailed solving method has been represented in Rowland et al. [34]:

(10) $${\mathrm{CL}}_{\mathrm{org}}=\frac{Q\cdot k_{\mathrm{el}}\cdot K_{P}\cdot V_{E}}{Q+k_{\mathrm{el}}\cdot K_{P}\cdot V_{E}}$$

in which CL_org_ denotes the organ clearance.

In Equation (10), k_el_K_P_V_E_ is defined as intrinsic clearance (CL_int_); in other words, an intrinsic capability of a liver to remove a drug from the blood without any flow limitations. The unit of k_el_K_P_V_E_ is identical to CL, and it is expressed by the following equation:

(11) $${\mathrm{CL}}_{\mathrm{int}}=k_{\mathrm{el}}\cdot K_{P}\cdot V_{E}$$

which from Equation (10) implies

(12) $${\mathrm{CL}}_{\mathrm{org}}=\frac{Q\cdot {\mathrm{CL}}_{\mathrm{int}}}{Q+{\mathrm{CL}}_{\mathrm{int}}}$$

Equation (12) indicates that CL_org_ is a function of Q and CL_int_. There are two circumstances depending on the relative size of the two variables.

1. The first situation is when the clearance capacity (i.e., CL_int_) exceeds the Q (CL_int_ >> Q). In this situation, Equation (12) collapses and transforms to Equation (13).
  (13) $${\mathrm{CL}}_{\mathrm{org}}≅Q,{\;\mathrm{if}\;\mathrm{CL}}_{\mathrm{int}}\gg Q$$
2. The second situation is when C_out_ is a small fraction of C_in_ (i.e., when K_p_ is high, or ER is low). [34]. In this case, Equation (12) collapses in the following equation:
  (14) $${\mathrm{CL}}_{\mathrm{org}}≅{\mathrm{CL}}_{\mathrm{int}},{\;\mathrm{if}\;\mathrm{CL}}_{\mathrm{int}}\ll Q$$

The basic assumption of the CL concept is that only an unbound free drug is accessible to the enzyme and is subjected to metabolism or biliary excretion. Therefore, the actual intrinsic clearance should be based on the unbound fraction in plasma (f_p_) or blood (f_b_). In practical settings, the calculation of protein binding and the analysis of drug concentration are usually performed with plasma. Interconversion between the free fractions in blood and in plasma is shown below:

(15) $$f_{b}=\frac{f_{p}\cdot C_{P}}{C_{B}}$$

(16) $$C_{B}=C_{\mathrm{RBC}}+C_{P}\left(1-H_{\mathrm{ct}}\right)$$

where C_B_ and C_P_ refer to the total drug concentration in blood and in plasma, respectively. H_ct_ is the hematocrit with a value of 0.44 in humans [35] and C_RBC_ refers to the drug concentration in red blood cells.

Therefore, CL_org_ is expressed by the equation below by incorporating f_p_:

(17) $${\mathrm{CL}}_{\mathrm{org}}=\frac{Q\cdot f_{p}\cdot {\mathrm{CL}}_{\mathrm{int}}}{Q+f_{p}\cdot {\mathrm{CL}}_{\mathrm{int}}}$$

#### 2.1.2. Consideration of Enzyme Kinetics

In Equation (12), if the clearing organ is the liver, the correlation between hepatic clearance (CL_H_) and enzyme kinetics is expressed by the equation below.

The metabolic rate (V_met_) in the liver is described by the Michaelis–Menten equation:

(18) $$V_{\mathrm{met}}=\frac{V_{\max}\cdot C}{K_{m}+C}$$

where V_max_ is the maximal rate of the reaction, C is the concentration of the substrate, and K_m_ is the Michaelis constant. If both sides of Equation (18) are divided by C, then V_met_/C is the hepatic intrinsic clearance (CL_int, H_) as shown in the following equation:

(19) $${\mathrm{CL}}_{\mathrm{int},H}=\frac{V_{\mathrm{met}}}{C}=\frac{V_{\max}}{K_{m}+C}$$

Since liver enzymes are rarely saturated in clinical practice, generally the value of K_m_ is much greater than C. Thus, Equation (19) can be simplified into the following equation:

(20) $${\mathrm{CL}}_{\mathrm{int},H}=\frac{V_{\mathrm{met}}}{C}=\frac{V_{\max}}{K_{m}}$$

Intrinsic clearance is also expressed by the summation of enzyme activities of all parallel metabolic pathways as shown in the following equation:

(21) $${\mathrm{CL}}_{\mathrm{int},H}=\overset{n}{\underset{i=1}{\sum }}\frac{V_{\max,i}}{K_{m,\;i}}$$

In an in vitro setting, the V_max_ and K_m_ are calculated. Then hepatic clearance is estimated by embedding the CL_int, H_ into Equation (12).

#### 2.1.3. Hepatic Clearance Model

Liver is one of the key organs for drug clearance via metabolism and/or excretion through the bile acid. For most drugs, the elimination process in PKs involves hepatic metabolism. Alteration of liver blood flow, synthesis of albumin, and/or enzyme activity could occur by liver impairment, concomitant drug use, environmental factors, and so on [36,37]. Therefore, predicting drug behavior in the liver facilitates the analysis of hepatic drug elimination in virtual scenarios [38].

In the field of PKs, there are four representative hepatic clearance models (Table 1).

##### Well-Stirred Model

The well-stirred model is a widely applied model, in which the liver is viewed as a single, well-mixed compartment with a fixed drug concentration. This model is expressed in simple equations.

CL_H_ is described by Equation (5):

CL_H_ = Q_H_· ER_H_ (22)

where Q_H_ is the hepatic blood flow (20.7 mL/min/kg in humans), and ER_H_ is the hepatic extraction ratio. Since ER is dependent on Q_H_, CL_H_ is not directly proportional to Q_H_. Typically ER decreases with increasing Q_H_ [32]. Additionally, hepatic availability (F_H_) is calculated by the following equation using ER_H_:

(23) $$F_{H}=1-{\mathrm{ER}}_{H}=\frac{Q_{H}}{Q_{H}+f_{p}\cdot {\mathrm{CL}}_{\mathrm{int},H}}$$

For the drugs with high ER_H_, equations of CL_H_, ER_H_, and F_H_ are simplified as the following equations:

(24) $${\mathrm{CL}}_{H}≅Q_{H}$$

(25) $${\mathrm{ER}}_{H}≅\frac{{\mathrm{CL}}_{\mathrm{int},H}\cdot f_{p}}{{\mathrm{CL}}_{\mathrm{int},H}\cdot f_{p}}{≅}_{1}$$

(26) $$F_{H}≅\frac{Q_{H}}{f_{p}\cdot {\mathrm{CL}}_{\mathrm{int},H}}$$

For the drugs with low ER_H_, the equations of CL_H_, ER_H_, and F_H_ are to be simplified to the following equations:

(27) $${\mathrm{CL}}_{H}≅{\mathrm{CL}}_{\mathrm{int},H}$$

(28) $${\mathrm{ER}}_{H}≅\frac{{\mathrm{CL}}_{\mathrm{int},H}\cdot f_{p}}{Q_{H}}$$

(29) $$F_{H}≅\frac{Q_{H}}{Q_{H}}{≅}_{1}$$

##### Parallel-Tube Model

The parallel-tube model describes the liver as a set of tubes representing a sinusoid where the elimination occurs in hepatocytes. Drug concentration within the liver (i.e., sinusoids and hepatocytes) exponentially decreases in the direction of the hepatic vein [39].

In this model, F_H_ is expressed by the following equation:

(30) $$F_{H}=1-{\mathrm{ER}}_{H}=e^{-\left(\frac{{\mathrm{CL}}_{\mathrm{int},H}}{Q_{H}}\right)}$$

When ER_H_ and Q_H_ are known, the CL_int,H_ is estimated by this model. Taking the natural logarithm of the Equation (30):

(31) $$\ln\left(1-{\mathrm{ER}}_{H}\right)=-\frac{{\mathrm{CL}}_{\mathrm{int},H}}{Q_{H}}$$

(32) $${\mathrm{CL}}_{\mathrm{int},H}=-Q_{H}\times \ln\left(1-{\mathrm{ER}}_{H}\right)=-Q_{H}\times \ln F_{H}$$

Both well-stirred and parallel-tube models assume that drug permeability is not a rate-limiting step in drug elimination [40]. However, recently, an extended clearance model has been developed in which permeability is one of the important factors affecting the CL_H_ [41,42].

In many cases, the well-stirred model is the choice of method for the estimation of CL_org_. However, in certain situations, the estimation of CL_H_ differs between the two models. Pang and Rowland have shown these differences [43,44,45]. In their studies, using lidocaine with an ER of 0.99 or higher, a liver perfusion experiment was conducted in mice. Its metabolite profile is well described by the well-stirred model. The major differences between these two models are F_H_ based on changes of Q_H_ and oral bioavailability (F_po_). When a drug with high ER_H_ (e.g., lidocaine) is administered via per oral (PO) route, its F_po_ is expressed by the following equation:

(33) $$F_{\mathrm{PO}}=F_{H}=1-{\mathrm{ER}}_{H}=e^{-\left(\frac{{\mathrm{CL}}_{\mathrm{int},H}}{Q_{H}}\right)}$$

Based on the well-stirred and parallel-tube model, the Equation (33) could be transformed into Equations (34) and (35), respectively:

(34) $$F_{\mathrm{PO}}≅\frac{Q_{H}}{f_{p}\cdot {\mathrm{CL}}_{\mathrm{int},H}}$$

(35) $$F_{\mathrm{PO}}≅e^{-\left(\frac{f_{p}\cdot {\mathrm{CL}}_{\mathrm{int},H}}{Q_{H}}\right)}$$

As shown in these equations, F_PO_ is associated with Q_H_. In a well-stirred model, F_PO_ shows a linear relationship with Q_H_. However, in the parallel-tube model, F_PO_ changes exponentially with Q_H_. By comparing the observed values with predicted values using these two models, the investigator can select the model that better explains the organ clearance. However, under practical experimental settings, it is hard to determine the model with a good fit prior to an investigation. Therefore, unless there is obvious evidence, most investigators use the well-stirred model based on the principle that models should be as simple as possible, but not simpler [40,46].

##### Distributed Model and Dispersion Model

It is obvious that the liver is neither a well-stirred compartment nor a series of identical tubes [47]. There have been efforts to explain hepatic clearance as more physiologically relevant by using a dispersion model [48,49] or a distributed model [50,51]. The distributed model describes the liver as a series of parallel tubes with different geometrical properties. In this model, ε^2^ is an estimated parameter used to express variance for each sinusoid in the whole liver [52]. In the distributed model, the mixing of blood in the sinusoids is incorporated into flow rates and path length. The degree of mixing is defined by the dispersion number D_N_ which is estimated in this model. When D_N_ → ∞ or D_N_ → zero, the dispersion model is collapsed in the well-stirred model and the parallel tube model, respectively. The variable ‘a’ in the dispersion model is equal to (1+4R_N_D_N_)^1/2^, where the efficiency number of R_N_ is equal to f_p_·CL_int,H_/Q_H_.

Other models presented by scholars include the series-compartment model [53] and transit-time model [54,55,56,57]. However, the IVIVE mainly uses the four models described above.

## 3. Prediction of Human Clearance Using IVIVE Method

### 3.1. IVIVE

**Figure 2:**
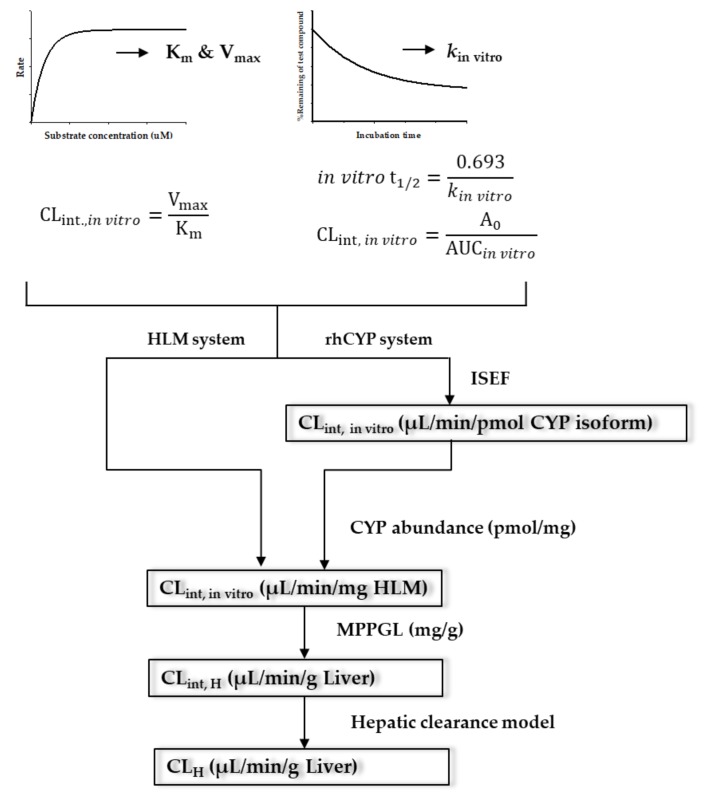
The scheme of the overall in vitro-in vivo extrapolation (IVIVE) process using human liver microsomes or recombinant human cytochrome P450 (CYP) system. MPPGL refers to the microsomal protein per gram of liver.

The purpose of IVIVE is to perform quantitative extrapolation of in vitro data to predict human parameters. A reliable extrapolation method to predict hepatic metabolic clearance utilizes in vitro kinetic data and mathematical equations [58]. The general approach of IVIVE using human liver microsomes (HLM) or recombinant human cytochrome P450 (CYP) system (rhCYP) is presented in Figure 2. Using these systems, metabolite production or substrate depletion are used to calculate the in vitro metabolic kinetic parameters (i.e., K_m_, V_max_, and k_in vitro_). The IVIVE method has been improved since its introduction by Rane et al. [59]. Scale-up of in vitro data to in vivo is performed by analyzing the correlation between in vitro and in vivo data or applying physiological correction factors. Many investigators have tried to improve the accuracy of prediction (Table 2).

#### 3.1.1. Empirical IVIVE Model

Scaling factors have been used to predict in vivo clearance from in vitro data. Correction factors are key components in this method. Various physiological or empirical values have been suggested in this approach. Appropriate scaling factors have been developed to improve the predictability of the IVIVE model. A direct physiological scaling factor was incorporated to predict CL_H_ using in vitro hepatocytes and rat microsomes data by Houston [60]. In that study, the basic principal and process of IVIVE were presented. The physiological scaling factor was investigated. Results indicated that this simple scaling factor yielded adequate evidence supporting IVIVE.

Another empirical analysis was performed by Lavé et al. [61], who used human hepatocytes as an in vitro system to predict human ER_H_. A scaling factor in Equation (41), shown in Table 2, was estimated using non-linear iterative least squares, which is not a fixed value. The predicted ER_H,pred_ and intrinsic in vitro clearance (CL_int, in vitro_) had a good relationship. In this method, no protein binding was considered, resulting in overestimation of ER_H,pred_ values of highly bound drugs. Nevertheless, the PK parameters of a few highly bound drugs, such as bosentan and lorazepam, were estimated with good agreement. The authors suggested that such discrepancy was attributed to the differences between the relative binding rate of the drug in the plasma and in hepatocytes, and/or its relative [61]. However, the overall predictability of human PK parameter was improved by applying a precise scaling factor, which plays a key role in the IVIVE method.

As shown in these results of Houston [46] and Lavé et al. [47], appropriate scaling factors are important in the IVIVE model to improve the predictability. Protein binding also has a critical impact on the prediction of in vivo PK parameters. The effect of binding properties on the prediction of CL has being investigated in other studies.

#### 3.1.2. Correction Factor of IVIVE Model

##### Protein Binding Factor

Obach [62] has reported the prediction method of human intrinsic hepatic clearance (CL_int, H, human_) using the in vitro half-life (t_1/2_) to incorporat non-specific binding factors to microsomes (f_u,mic_) and/or the f_p_. Twenty-nine drugs were classified according to their chemical property (i.e., basic, neutral, and acidic compounds). Generally, the basic compounds tend to have a large extent of binding. Results showed that human CL of neutral and basic compounds was adequately predicted with or without binding factors. However, in case of acid compounds, excluding binding factors, human CL values were predicted with a high degree of error.

In practice, in the absence of prior PKs and/or ADME knowledge of a compound of interest, one cannot easily decide whether or not to consider protein binding when predicting human PK parameters. Therefore, the projection of human CL considering both binding factors (i.e., in vitro microsomes binding and the fraction unbound in plasma) is a strategy to decrease significant risks of over/under estimation of human CL while expanding the predictability.

The effect of microsomal protein binding on the prediction of CL_int_ was also investigated by Austin et al. [63]. In their work, rat liver microsomes were used as an in vitro system. Their results showed that the CL_int_ was dependent on microsomal concentration. However, this relationship can be ignored when f_u,mic_ is considered. The authors also found that f_u,mic_ was correlated with lipophilicity. Based on these results, the authors formulated an equation for the calculation of f_u,mic_ based on the physicochemical properties of drugs. Equation (36) can be used to calculate f_u,mic_ as follows:

(36) $$f_{u,\mathrm{mic}}=\frac{1}{C\times 10^{0.56\left(\log\;P/D\right)-1.41}+1}$$

where C denotes the microsomal protein concentration (mg/mL) and log P/D refers to logP of a basic compound (pKa > 7.4) or logD7.4 of acidic compound (pKa < 7.4), where logD_7.4_ stands for the partition coefficient between octanol-0.02 M phosphate buffer (pH 7.4 at 20 °C). The logP is equal to the logD_7.4_ for compounds designated as neutral and the logP is also calculated using the following equation:

(37) $$\mathrm{logP}={\mathrm{logD}}_{7.4}+\log\left(1+10^{7.4A+{\mathrm{BpK}}_{a}}\right)$$

where A = 1 and B = −1 for an acidic compound, and A = −1 and B = 1 for a basic compound [64].

Howgate et al. [65] revealed that most of the f_u,mic_ values are high enough to be ignored in the prediction of clearance. However, the few compounds with high microsomal binding should be considered to accurately predict the in vivo clearance. Therefore, when basic knowledge of the compound of interest is lacking in the early stage of drug discovery and development process, incorporating f_u,mic_ is a preferable way to predict in vivo situations.

##### Animal Scaling Factor

Naritomi et al. [66] recommended the IVIVE method of the animal scaling factor, which is defined as CL_int, in vivo_ divided by CL_int, in vitro_ to improve the human CL_int, in vivo_. This scaling factor is similar across species, since it depends on the compound itself. When the animal scaling factor in a rat or a dog was not considered, the average fold error increased (from an average two-fold to four-fold error). These results indicate that the scaling factor of each drug is conserved across an inter-species system. However, an animal scaling factor is difficult to use in the absence of adequate information for various species.

#### 3.1.3. Inter-Individual Variability (IIV) in the IVIVE Method

The prediction of human CL_H_ by IVIVE is generally limited by IIV, most likely due to drug metabolizing enzymes [74]. Several studies have reported the substantial differences in CYP expression and significant differences in the activity of different CYP isoforms in HLM [68,75,76]. The potential variation in the abundance of protein expression in relevant organs can be incorporated into IVIVE [77].

The microsomal protein per gram of liver (MPPGL) value can be used as a scaling factor to calculate CL_int, in vivo_ from CL_int, in vitro_. Generally, a value of 45 mg/g liver [60] originally obtained from rat data, or 52.5 mg/g based on hepatocyte data reported in the literature via back calculation, is commonly used as MPPGL. Since the pharmacogenetic data of laboratory animal models are less than those of humans because of their genetics and environment, the variation in MPPGL of humans may be greater than that of rats [78].

##### Microsomal Protein Content and CYP Abundance

Since a maximum limit for microsomal CL may exist [59], drugs with high CL tend to have under-predicted CL_int, in vivo_ if data are derived from microsomal protein [51]. Carliel et al. [67] have investigated diazepam as a model drug with high clearance. Its CL_int, in vivo_ is 160 mL/min/SRW, where SRW refers to standard rat weight of 250 g. Microsomal content was adjusted by treating phenobarbital and dexamethasone as CYP inducing agents. The scaling factor calculated from Equation (49) was used to estimate CL_int, in vivo_. The results showed a good agreement with observed in vivo clearance. Although a CL_int, in vitro_ obtained from dexamethasone-treated microsomes provided an accurate estimate of 77% of the observed CL_int, in vivo_, the limitation similar to that of Houston [60] persisted. The relationship between CL_int, in vitro_ and CL_int, in vivo_ was investigated empirically rather than mechanistically. Nonetheless, this study suggested that variation in CYP content affects the prediction of in vivo clearance. It provides evidence supporting the incorporation of CYP content as a covariate affecting the IIV in the IVIVE model.

Correction factors of both microsomal protein content and CYP abundances have been included in the IVIVE method using the f_u,mic_ factor by Howgate et al. [65]. Underestimation of the parameter is a general issue in the IVIVE method. Inclusion of the microsomal protein content and CYP abundances that affect the IIV did not show the trend of underestimation.

##### Microsomal Protein per Gram of Liver (MPPGL)

MPPGL is a key value for the scaling of CL_int, in vitro_ to CL_int, in vivo_ using liver microsomes data or the rhCYP system as shown in Figure 2. It is a value with varying degrees of IIV. However, investigators have been using fixed values either due to the lack of information or empirically.

Barter et al. [78] have reported MPPGL variability via meta-analysis and have investigated potential covariates affecting MPPGL [79]. In their studies, the authors reported an inverse correlation between age and MPPGL. The MPPGL values range from 40 mg/g and 31 mg/g for those in their 30s and 60s, respectively. The authors also provided the following equation to calculate age-related values of MPPGL from birth to adult:

(72) $$\mathrm{MPPGL}\;\left(\mathrm{mg}/g\right)=10^{(1.407+0.0158\times \mathrm{age}-0.00038\times {\mathrm{age}}^{2}+0.0000024\times {\mathrm{age}}^{3}}$$

The results provide key information to project PK parameters to humans, especially prior to clinical trials. Healthy subjects constitute the typical population for a clinical pharmacology study during the early phase of drug development, whereas real world patients are very disparate. Estimation of the PK parameters of special populations (e.g., pediatric or geriatric patients) is one of the challenging tasks in clinical trials. Of course, various factors that affect PKs in a special population have been studied. These results provide meaningful insight suggesting that non-clinical data may be considered for the design of clinical trials representing special populations.

##### Inter-System Extrapolation Factor (ISEF)

The use of a recombinant system represents an alternative in vitro method instead of human liver samples, for prediction of in vivo metabolic clearance. Iwatsubo et al. [68] have suggested the use of CYP450 isoform content in the recombinant method and proposed a P450 content correction factor. Since the levels of CYP450 reductase and cytochrome b5 differ from those of human livers (lower, in most case) in a recombinant system, the authors have proposed an additional correction factor, expressed in Equation (51). The authors have concluded that the prediction of in vivo CL using recombinant system is possible if metabolic activity is corrected for the CYP isozyme content both in rhCYP and per gram liver in vivo.

ISEF, which is a dimensionless value based on the activity of CYP isoform and its contents, has been defined by Proctor et al. [70]. It is used to direct scale data from a rhCYP system to an HLM environment for evaluation of differences in intrinsic activity (per unit CYP) and IIV by incorporating CYP abundance as shown in Equations (52) to (56). Population approached IVIVE could be performed by combining the variance in physiological parameters (such as liver blood flow and liver weight) and the variance in scaling parameters (such as MPPGL and ISEF).

Nakajima et al. [69] have suggested a modified version of the relative activity factor (RAF) using CL_int, in vitro_ to correct a flaw in the original RAF, which was calculated with V_max_ alone while the K_m_ value was ignored. In their study, RAF represents the ratio of CL used to predict clearance of azelastine. It best reflects observed N-demethylation CL in HLM.

Chen et al. [80] have experimentally determined the ISEF of six CYP isoforms and investigated their utility in early phases of drug discovery and development. Venkatakrishnam et al. [81] have also investigated the role of CYP1A2, CYP2B6, CYP2C19, CYP3A, and CYP2D6 in a lymphoblastic cell line and suggested the incorporation of bridging factors between rhCYP and liver microsomes, such as RAF and abundance of CYP isoform in microsomes.

#### 3.1.4. Additional Correction Factors

In the conventional IVIVE method, the prediction of in vivo CL using in vitro metabolic data has been performed with good agreement. However, this method could not be used for drugs with high binding rate to plasma and/or blood protein, low CL, or high interaction with transporters. To overcome these limitations, recently, investigators have tried to develop new IVIVE methods using physiologically-based and mechanistic approaches which are presented below in detail.

##### F_I_

Only unbound and unionized forms of drugs have access to hepatocytes, which are the sites of metabolism. In the conventional IVIVE model, protein binding is a key factor contributing to the accuracy of CL prediction. Berezhkovskiy [71] has developed a modified equation to predict CL_H_ based on differences in intra- and extra-cellular pH of the unbound drug fraction using F_I_ as presented in Equations (57) to (60). These equations yielded higher values (up to 6.3-fold) of CL_H_ for a basic compound (F_I_ > 1) for strong diprotic bases, but lower values (up to 6.3-fold) of CL_H_ for an acidic compound (F_I_ < 1) for strong diprotic acids. The author suggests that the modified equation with F_I_ improved the issue of both under- and over-estimation commonly encountered in IVIVE. Therefore, for basic compounds, the modified equation could improve the prediction of CL_H_. For acidic drugs, the conventional IVIVE equation tends to overestimate the CL_H_. However, this modified equation also improves the prediction of CL_H_ for acidic compounds. Especially, the ionization factor significantly influences the calculation of CL_H_ for drugs with a low extraction ratio since CL_H_ is directly proportional to F_I_ in this case.

##### Effective Fraction Unbound in Plasma

Calculation of drug concentration in the site where metabolism occurs is important in IVIVE methodology since only free-form drugs penetrate the cellular membrane to reach the metabolic enzyme. Ionic interactions between extracellular binding proteins and the hepatocyte surface provide higher cellular exposure for the unbound drugs than without consideration of the interactions [82].

Poulin et al. [72,83,84] have developed a mechanistic IVIVE model based on two additional factors including pH differences between extracellular and intracellular water in liver, and protein-facilitated uptake induced by potential ionic interactions between protein-albumin bound drug complex and cell surface. This mechanistic IVIVE model overcomes the prediction of human CL for drugs with low CL_int_ and high binding affinity for proteins commonly encountered when predicting human CL from in vitro data. Equations suggested by Poulin presented in Equations (61) to (62), incorporate the new correction factor of unbound fraction in the liver.

#### 3.1.5. Physiologically-Based IVIVE Model

Despite several attempts to accurately predict drug concentrations in the liver where metabolism takes place, the comparative analysis from Hallifax and Houston [85] reported fewer differences in accuracy for the prediction in vivo CL, calculated by Berezhkovskiy and Poulin, using conventional methods. Furthermore, the authors have underscored the need to develop a model that reflects additional physiological factors and mechanistic elucidation to overcome the limitations of existing methods.

In the disposition process, transporters and enzymes play a key role by interacting with each other [86]. Conventionally, IVIVE methods are focused on a single pathway of drug metabolism. However, a drug introduced into the body is cleared via the ADME pathways, which involves numerous enzymes and transporters.

Wu et al. [87] have suggested a Biopharmaceutics Drug Disposition Classification System (BDDCS), which is modified by the Biopharmaceutics Classification System based on routes of drug elimination and the effect of efflux and absorptive transporters. Their study revealed that highly permeable compounds are highly metabolized whereas less permeable compounds tend to be eliminated via renal and/or biliary excretion in intact form.

A novel IVIVE method was developed to predict hepatic organ clearance via physiology-based modeling [42,73] as shown in Equations (63) to (64). This new method reflects additional physiologically relevant information (namely hepatic uptake, metabolism, biliary excretion, and sinusoidal efflux) compared with the conventional method. The proposed method was used to predict rat hepatic clearance of 13 compounds with various physicochemical and PK characteristics. In these studies, the hepatic clearance of valsartan (class 2 compound based on BDDCS) was underestimated with the highest fold-error of 3.95. The rate-limiting steps of class 2 compounds include metabolism and biliary excretion. Although this method incorporates both biliary excretion and metabolism in a typical single parameter prediction, underestimated cases such as valsartan prevailed probably due to its high plasma protein binding (97%) feature. However, since plasma protein binding is considered in the model, the error might have occurred due to unknown non-hepatic elimination.

Although these novel IVIVE methods provide precise prediction and detailed information of CL, additional in vitro data are required compared with the conventional single parameter prediction. Furthermore, in the early phases of drug discovery and development, it may be difficult to apply high-throughput screening, which is an advantage under in vitro experiment settings. However, this novel IVIVE method represents a very useful tool for the evaluation of optimized candidates prior to clinical trials.

An extended clearance model (ECM) based on hepatobiliary clearance has been reviewed by Camenisch et al. [41]. The same group proposed a new IVIVE method for the prediction of total clearance for accurate prediction of relative elimination contribution. Two mathematical Equations (66) and (67) depict the relationship between PS_inf_ and fn_H_; and PS_inf,pas_ and fn_met_. In vitro data (i.e., hepatic uptake data) based on suspensions of human hepatocytes fn_H_ and fn_met_ can be calculated using the equations.

Along with estimated fractional parameters, total clearance can be presented as the sum of parallel connected organ clearances assuming the absence of extra-hepatic and/or renal clearance. This practical method facilitates the determination of the mechanism of elimination pathway using only in vitro data.

## 4. Application of AS for the Prediction of Human PK Parameters

### 4.1. Concept of AS

Allometry is the study of the relationship between size and physiological parameter. It is the study of the usual variation in measurable characteristics of anatomy and physiology as a function of overall body size [88]. The allometric equation is generally expressed as a power function based on the following equation [20]:

(73) $$Y={\mathrm{aW}}^{b}$$

In Equation (73), the Y and X represent quantitatively measurable variables, a denotes constant of appropriate unit, and b is a power exponent. In the PKs, Y is a parameter of interest and B is a physiological parameter, and W is weight. In general, a is drug dependent and b is parameter-type dependent, which are approximately 0.75 for CL and chemical-specific factors associated with metabolism (e.g., V_max_), 1 for volume of organs and blood flows, and 0.25 for physiological times [18,88]. AS assumes that mammals share similar anatomical, biochemical, and physiological features [16,21].

### 4.2. Prediction of Clearance by AS

There have been various attempts to predict human clearance. Mathematical equations are presented in Table 3. Since simple AS is one of the simplest methods for prediction of human PK parameter, it is widely used for scale-up of prediction from non-clinical PK data to humans. Although it is simple and useful by nature, simple AS has not been entirely successful for the prediction of human clearance. To overcome this limitation, various groups have reported new methods of AS. 

#### 4.2.1. Two-Term Method

Boxenbaum and Fertig [17] have developed the two-term method. In their work, the intrinsic clearance of antipyrine was predicted using a two-term allometric equation including brain weight (BW) and weight (W) based on Equation (77). The equation was in good agreement with the relationship between antipyrine intrinsic clearance and physiological variables, BW and W. However, in their article, only a single drug, antipyrine, was tested. Further investigation revealed that this two-term equation was limited to general conditions.

#### 4.2.2. Rule of Exponent

Mahmood and Balian [6,89,101,102,103,104,105,106] have contributed to numerous works on AS. In their studies, they compared the CL value of antiepileptic drugs using four different allometric equations: a simple AS in Equation (74), the product of CL and MPL in Equation (75), the Boxenbaum’s two-term power function in Equation (77), and the product of CL and BW in Equation (76), a novel equation developed by Mahmood and Balian.

It is well known that the simple AS adequately predicts the CL of a drug, which is mainly cleared by renal excretion. However, under general circumstances, simple AS was not adequate to predict CL. When Boxenbaum’s two-term method was used to predict the CL of antiepileptic drugs, the prediction failed. When MLP and brain weight were incorporated in simple AS, the predicted values showed good agreement with the observation values [89].

Mahmood and Balian extended their studies to another work [105] by applying three of four AS methods (except for the two-term power method) to drugs with various physiochemical and PK properties.

Moreover, the ROE, established by Mahmood and Balian [105], provides a guide for the selection of appropriate AS methods for the prediction of drug clearance. The choice of method depends on the exponent determined by simple AS. If the exponent lies between 0.55 to 0.7, the simple AS method is reasonable. If the exponent lies between 0.71 to 1.0, clearance can be predicted reasonably well using CL product MLP method. If the exponent is larger than 1.0, clearance can be predicted using CL product BW method [105]. Mahmood [103] also tested the ROE to predict oral clearance by the same approach. Results showed that the ROE also predicted oral clearance.

Some researchers have expressed concerns about AS since correction factors such as MLP and BW have no clear biological rationale [107]. The fact that three or more species are needed for a reliable prediction of CL [108] is time consuming and costly in the drug development process. However, considering that various experiments are conducted during the drug discovery and development process, at least two animals in an in vivo study are necessary for the non-clinical study [109].

The ROE method could be applied to predict a human CL for biliary excreted drugs. Correction factors are derived based on bile flow with normalization by body weight and liver weights. After the appropriate method is selected by ROE, the CL in a given species is divided by the calculated bile flow correction factor and scaled. The predictability of human CL is significantly improved using the bile flow correction factor [110].

#### 4.2.3. One or Two Species Method

A few studies have reported AS using only one or two species [90,94,95]. As presented in Equations (80) to (85), these empirically determined equations provide valuable information about predicted human CL. Results using these methods are in good agreement. Especially, considering the limited available data in the early phase of drug discovery and development process, these methods are useful tools for the prediction of human CL and provide evidences for go or no-go decision-making.

#### 4.2.4. Liver Blood Flow

Liver blood flow is used as a correction factor in AS. Liver blood flow is used to predict human CL using Equation (86). It has been suggested that the simple liver blood flow-based scaling is the best method and that monkey liver blood flow (MLBF) is superior to predict human CL from rats, dogs, and monkeys’ CL data [96].

The advantage of this method is clear in that it only needs a single species to scale up from animal data. In addition, the MLBF method is particularly applicable to drugs that are not readily metabolized and/or renally excreted when administered intravenously [101]. However, Mahmood [101] has raised an issue about the MLBF method reported by Nagilla and Ward [96]. Mahmood [101] claimed that the reported MLBF method had statistical flaws and that the dataset used in their work [96] should be clarified. Furthermore, the bile flow used in the study did not match with the bile flow rate reported by Davies and Morris [35] which they cited. Furthermore, the MLBF method assumes that data from rats, dogs, and monkeys are always available. However, this is not true. In addition, this method is based on only three species.

#### 4.2.5. Incorporation of in Vitro Data

Lave et al. [93] have investigated in vitro data with AS to predict hepatic clearance for 10 drugs that are extensively metabolized. They determined the rate of metabolism in various animal species via in vitro experiments, including human liver microsomes and hepatocytes. The authors concluded that correcting clearance with in vitro metabolic rates significantly improved the prediction of human CL compared with direct scaling or correction with BW.

Although this in vitro correction method provided a rationale based on physiological factors, Mahmood [102] demonstrated that MLP corrected AS produced the same results. However, this in vitro correction method showed a clear disadvantage in that in vitro CL from several species must be determined in the MLP correct AS method, which is time consuming and costly [22].

#### 4.2.6. Protein Binding

Theoretically, only unbound drugs can be distributed to the hepatocytes where the metabolism occurs and/or kidney excretes. Since the protein binding properties of a drug vary between species [111], disposition of the drug may be variable in different species. Chiou et al. [112] have reported the effect of protein binding on prediction of human clearance in AS for 15 extensively metabolized drugs. In their work, consideration of protein binding to correct inter-species differences in AS tended to improve the prediction of human clearance. Although it is theoretically feasible to use AS with unbound clearance (CL_u_ = CL/f_u_) based on protein binding, in practice, f_u_ does not significantly improve its predictability [22], [113].

Mahmood [104] has investigated the role of protein binding in the prediction of CL using 20 randomly selected drugs. Furthermore, Mahmood [6] has compared total CL and CL_u_ and found that for drugs excreted renally or via extensive metabolism, CL_u_ could not be predicted any better than total CL.

#### 4.2.7. QSAR Approach

Wajima et al. [98,99] have tried to predict human CL based on physicochemical properties of drugs. In their method, human CL was predicted using descriptors including MW, cLogP, and Ha. Observed rat and dog data were incorporated into their analysis. Their method facilitated prediction of human clearance.

#### 4.2.8. Fraction Unbound Intercept Correction Method (FCIM)

FCIM was developed by Tang et al. [97] for prediction of human CL. In this method, water-octanol partition coefficient and the ratio of f_p_ between rats and human (Rf_u_) are considered. The authors concluded that the new method significantly improved the prediction, even better than ROE. Furthermore, this method improved the prediction of vertical allometry.

However, when Mahmood [106] performed comparative analysis using ROE and FCIM for drugs with various PK properties (i.e., extensively metabolized, renally excreted and/or secreted and biliary excreted), the results showed that both methods facilitated the prediction of human clearance. In some cases, one of these two methods could be more suitable for predictions. However, the author expressed concern about FCIM since it uses a fixed exponent of 0.77 and a constant of 33.35 while exponents of AS are dependent on the species used in the scaling. Furthermore, FCIM is not suitable for renally secreted and biliary excreted drugs. Despite such concerns, when both methods are considered, it is possible to predict CL in a wide range of drugs.

#### 4.2.9. Multiexponential Allometric Scaling (MA)

Goteti et al. [92] have developed a new method for animal scale up using MA. In this method, the human CL is estimated by the equation below:

(90) $$\mathrm{CL}={\mathrm{aW}}^{b}+{\mathrm{cW}}^{d}$$

where a and b represent coefficient and exponent obtained from simple AS, respectively, and c and d are coefficient and slope from MA, respectively.

The slope of MA (i.e., the value of d in Equation (90)) is determined by plotting blood flow rate, organ volume, and organ weights of liver and kidney in non-clinical species against W. As a result, the slopes of liver and kidney were found very similar. The value of d is fixed as 0.9. The coefficient of c is the function of the coefficient of a from AS. The final MA equation is derived as shown in Equation (78).

The MA method can successfully predict human clearance. Their results indicate that monkey is an important species for scaling. When the exponent of simple AS was greater than 0.7, MA showed better prediction of human CL than the simple AS method.

### 4.3. Prediction of Volume of Distribution by AS

#### 4.3.1. Volume of Distribution in PKs

There are three types of volume of distribution (V_d_) and generally estimated in PKs.

- Volume of distribution of central compartment (V_c_).
- Volume of distribution at steady state (V_ss_)
- Volume of distribution by area (V_area_), also known as V_β_

V_c_ is used as a correlation factor for the concentration and number of drugs in the body by the following equation:

(91) $$X=V_{c}\times C$$

where X and C refer to the amount of drugs in the body and concentration in the blood, respectively. Of these three types of volume of distribution, V_c_ is generally predicted from animal data. Its predictability is better than the others [114].

The following equations show that V_d_ is clearly different from the actual tissue volume where drugs are distributed in the body:

(92) $$\begin{array}{l} X=V_{\mathrm{bl}}\cdot C+\overset{n}{\underset{i=1}{\sum }}V_{i}\cdot C_{i}\\ =V_{\mathrm{bl}}\cdot C+\overset{n}{\underset{i=1}{\sum }}V_{i}\cdot K_{i}\cdot C\\ =\left(V_{\mathrm{bl}}+\overset{n}{\underset{i=1}{\sum }}V_{i}\cdot K_{i}\right)\cdot C\\ =V_{d}\cdot C \end{array}$$

where V_bl_ is the volume of blood, V_i_ is the volume of organ, C_i_ is the concentration in the organ, and K_i_ is the partition coefficient (K_i_ = C_i_/C). In this equation, the greater the tendency to distribute to tissues from blood (i.e., the greater K_i_), the greater is the V_d_.

#### 4.3.2. Prediction of V_d_

Various methods have been developed to predict V_d_, and the equations are presented in Table 4. In general, V_d_ is well correlated with body weight, indicating that the exponent of V_d_ is around 1 (usually between 0.8 and 1.1) [115]. Furthermore, for the prediction of V_d_, the two species in AS are acceptable compared to the use of three or more species. In the study of Mahmood and Balian [108], the average exponents using the simple AS for the prediction of V_d_ are 0.89 and 0.90 in case of 3 and 2 species, respectively.

The effect of protein binding on the prediction of V_d_ by AS has been investigated. As mentioned above, it is well known that protein binding properties vary between species. Furthermore, only unbound drugs penetrate blood vessels and biological membranes. For a drug with low binding affinity to plasma and tissue protein or drugs that are only distributed in the extracellular space, they can be scaled since total body water and extracellular water shows inverse correlation with animal size in AS [116].

Sawada et al. [117] have reported that considering the unbound fraction in the prediction of V_d_ may increase the accuracy of prediction results than the volume against unbound fraction in the plasma. In another study of Sawada et al. [118], the authors investigated the prediction of disposition of beta-lactam antibiotics and reported large differences in free volume of distribution between species. However, additional work revealed no advantage in consideration of the unbound fraction when Sawada et al.’s work was re-evaluated by adding six more drugs from the study of Mahmood [115].

#### 4.3.3. Prediction of Elimination Half-Life by AS

Elimination half-life (t_1/2_) is one of the most important PK parameter determining the dosage regimen and drugability. Predicted CL cannot estimate the t_1/2_ since the V_d_ and CL are required for the estimation of t_1/2_ as presented by the equation:

(98) $$t_{1/2}=\frac{0.693V_{d}}{\mathrm{CL}}$$

Because of the hybrid nature of the t_1/2_, this parameter has been poorly estimated by AS [89,114]. Instead of direct scaling of t_1/2_, Mahmood [89] has suggested the calculation of t_1/2_ as a secondary parameter using Equation (98). Another approach for prediction of human t_1/2_ is based on the mean residence time (MRT) [114]. The MRT represents the average staying time of the drug in a body organ or compartment as the molecules diffuses in and out [28] and the parameter is estimated by the following equation:

(99) $$\mathrm{MRT}=\frac{V_{\mathrm{ss}}}{\mathrm{CL}}$$

where CL is calculated by the Equation (1). Since V_ss_ is the summation of the volume of the central compartment (V_c_) and peripheral compartment (V_p_) in a two-compartment PK model, MRT can also be expressed with the equation below combined with Equation (2) [28]:

(100) $$\mathrm{MRT}=\frac{V_{c}+V_{p}}{k\cdot V_{c}}$$

Mahmood [114] investigated the prediction of MRT by AS. Results showed good agreement. Therefore, the t_1/2_ was predicted using the predicted MRT by following equation:

(101) $$t_{1/2}=\frac{\mathrm{MRT}}{1.44}$$

## 5. Prediction of Absorption Related PK Parameters

Absorption rate constant (k_a_) is generally expressed by first or zero order constant. It could be estimated from various PK models. However, k_a_ is originally an apparent parameter that can be best estimated through first-order loss of drug from the gastrointestinal tract, not through first-order appearance of drug in the plasma [52].

AS was applied to predict turn-over parameters. Turn-over rate refers to the amount that a compound is secreted or synthesized per unit time [52]. Therefore, in general, neither is ka scaled from animal data, nor is the affinity parameter (i.e., K_m_) in Michaelis-Menten equation applied by body weight scaling. [88]. Although AS equation for scaling the first order kinetic parameter (i.e., k) has been suggested by Kenyon [88] as shown in Equation (102) in Table 5, further evaluation is required.

Various empirical relationships of effective permeability (P_eff_) with physicochemical properties or Caco2 in vitro data have been reported. They are shown in Table 5. The k_a_ was estimated with the predicted P_eff_ combined with Equation (112). Another way to predict k_a_ is to use the mean value of absorption parameters from animal data. Liu et al. [123] have reported a method for human PK projection of imigliptin using IVIVE, AS, and PK/PD modeling. In their study, the absorption parameter was applied as the mean value in non-clinical animal models such as rats, dogs, and monkeys.

Equations for predicting the fraction of absorption (F_a_) have been reported by a few investigators. In Equation (115), F_a_ is predicted by a mechanism-based model using equilibrium solution for k_a_. Other relationships between F_a_ and P_eff_ are presented in Equation (116). The empirical equation could be used for prediction of F_a_ using in vitro permeability data.

## 6. Conclusions

Investigating the CL pathway is a significantly important issue in drug development. Drug CL parameters have an impact on the determination of dosing regimens for both normal and special populations, such as pediatric, elderly, and patients with renal or hepatic impairment, drug–drug interactions, and so on.

Prediction of PK parameters from non-clinical studies is essential in the drug discovery and development process. Over the last five decades, numerous translational approaches have been developed to predict human PK parameters. Both IVIVE and AS methods provide insight based on non-clinical studies for decision-making in the drug discovery and development process.

In the overall prediction of total clearance, AS represents a powerful method for the use of non-clinical data from single or multiple species. However, it is difficult to determine the variation in transporters and/or enzyme expression, affinity, and specificity with AS.

The proposed ECM model combined with the prediction of contribution could represent a breakthrough in AS and conventional IVIVE methodology.

Integration of the in vitro data and in vivo animal data is recommended for accurate prediction of specific ADME processes in humans [7] and recently, the combined methods were applied to a drug development process [123,131]. Since the choice of method depends on data availability and each method has advantages and disadvantages, the designing of an overall non-clinical study to generate appropriate data for scaling is one of the key steps in the practice of investigation.

Despite its uncertainty, ongoing refinement of IVIVE and AS methods will increase the accuracy of predictability and increase our understanding of the underlying rationale into mechanisms of extrapolation from in vitro or in vivo to human.

## Figures and Tables

**Table 1:** Four hepatic clearance models.

Model	Scheme ^1^	CL_H_^2^	ER_H_
**Well-stirred**	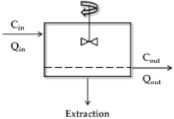	$\frac{Q_{H}\cdot {\mathrm{CL}}_{\mathrm{int},H}\cdot f_{p}}{Q_{H}+{\mathrm{CL}}_{\mathrm{int},H}\cdot f_{p}}$	$\frac{{\mathrm{CL}}_{\mathrm{int},H}\cdot f_{p}}{Q_{H}+{\mathrm{CL}}_{\mathrm{int},H}\cdot f_{p}}$
**Parallel tube**	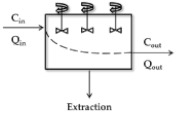	$Q_{H}\cdot \left\{1-e^{-\left(\frac{f_{p}\cdot {\mathrm{CL}}_{\mathrm{int},H}}{Q_{H}}\right)}\right\}$	$1-e^{-\left(\frac{f_{p}\cdot {\mathrm{CL}}_{\mathrm{int},H}}{Q_{H}}\right)}$
**Distributed**	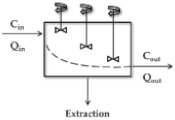	$Q_{H}\cdot \left\{1-e^{-\left(\frac{f_{p}\cdot {\mathrm{CL}}_{\mathrm{int},H\;}}{Q_{H}}+\frac{1}{2}\cdot \varepsilon^{2}{\left(\frac{f_{p}\cdot {\mathrm{CL}}_{\mathrm{int},\;H}}{Q_{H}}\right)}^{2}\right)}\right\}$	$1-e^{-\left(\frac{f_{p}\cdot {\mathrm{CL}}_{\mathrm{int},H}}{Q_{H}}+\frac{1}{2}\cdot \varepsilon^{2}{\left(\frac{f_{p}\cdot {\mathrm{CL}}_{\mathrm{int},H}}{Q_{H}}\right)}^{2}\right)}$
**Dispersion**	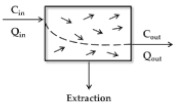	$Q_{H}\cdot \left\{1-\frac{4a}{{\left(1+a\right)}^{2}\cdot e^{\left[\frac{a-1}{2D_{N}}\right]}-{\left(1-a\right)}^{2}\cdot e^{-\left[\frac{a+1}{2D_{N}}\right]}}\right\}$	$1-\frac{4a}{{\left(1+a\right)}^{2}\cdot e^{\left[\frac{a-1}{2D_{N}}\right]}-{\left(1-a\right)}^{2}\cdot e^{-\left[\frac{a+1}{2D_{N}}\right]}}$

^1^ Dotted line indicates the concentration–distance profile within liver. ^2^ Where Q_H_ is hepatic liver flow expressed as a unit of mL/min/kg.

**Table 2:** Mathematical equations of the IVIVE approach for prediction of clearance from in vitro data.

Equation		Comment *	Ref.
${\mathrm{CL}}_{\mathrm{int},in\;vitro}=\frac{V_{\max}}{K_{m}}=\frac{\mathrm{rate}\;\mathrm{of}\;\mathrm{metabolism}}{C_{E}}$	(38)	Basic principle of IVIVE was suggested Provide the 4 stages for the IVIVE	[60]
${\mathrm{CL}}_{H,in\;vivo}=\frac{Q_{H}\cdot f_{b}\cdot {\mathrm{CL}}_{\mathrm{int},in\;vivo}}{Q_{H}+f_{b}\cdot {\mathrm{CL}}_{\mathrm{int},\mathrm{in}\;\mathrm{vivo}}}{\;\mathrm{or}\;\mathrm{CL}}_{\mathrm{int}}=\frac{{\mathrm{CL}}_{H}}{f_{b}\left(1-E\right)}$	(39)		
${\mathrm{CL}}_{\mathrm{int}.in\;vitro}=\frac{\mathrm{Initial}\;\mathrm{amount}\;\mathrm{in}\;\mathrm{the}\;\mathrm{incubation}}{{\mathrm{AUC}}_{in\;vitro}}$	(40)	Empirically the scaling factor (SF) was estimated as the value of 8.9 Predicted ER_H_ and observed ER_H_ are ER_H, pred_ and ER_H, obs_, respectively Provide criteria for the classification of the drugs into: low extraction, ER_H_ < 0.3; intermediate, 0.3 < ER_H_ < 0.7; high extraction, ER_H_ > 0.7	[61]
${\mathrm{ER}}_{H,\mathrm{pred}}=\frac{\mathrm{SF}\cdot {\mathrm{CL}}_{\mathrm{int},in\;vitro}}{Q_{H}+\left(\mathrm{SF}\cdot {\mathrm{CL}}_{\mathrm{int},in\;vitro}\right)}$	(41)		
${\mathrm{ER}}_{H,\mathrm{obs}}=\frac{\mathrm{CL}}{Q_{H}}$	(42)		
${\mathrm{CL}}_{\mathrm{int},H,\mathrm{human}}=\frac{0.693}{in\;vitro{\;t}_{1/2}}\cdot \frac{\mathrm{mL}\;\mathrm{incubation}}{\mathrm{mg}\;\mathrm{microsomes}}\cdot \frac{45\;\mathrm{mg}\;\mathrm{microsomes}}{g\;\mathrm{liver}}\cdot \frac{20\;\mathrm{mg}\;\mathrm{liver}}{\mathrm{kg}\;\mathrm{body}\;\mathrm{weight}}$	(43)	Investigation of the effect of the protein binding into the plasma and microsomes The ISTD refers to the internal standard	[62]
fu,mic=drugISTDpeak height ratio in buffer sample2·drugISTDpeak height ratio in microsome sample	(44)		
${\mathrm{CL}}_{\mathrm{int},H,\mathrm{pred}.}={\mathrm{CL}}_{\mathrm{int},in\;vitro}\cdot \mathrm{animal}\;\mathrm{scaling}\;\mathrm{factor}$	(45)	Animal scaling factor was incorporated into IVIVE	[66]
$\mathrm{Animal}\;\mathrm{Scaling}\;\mathrm{factor}=\frac{{\mathrm{CL}}_{\mathrm{int},H,in\;vivo}}{{\mathrm{CL}}_{\mathrm{int},in\;vitro}}$	(46)		
$f_{u,\mathrm{mic}}=\frac{\mathrm{unchanged}\;\mathrm{compound}\;\mathrm{concentration}\;\mathrm{in}\;\mathrm{buffer}}{\mathrm{unchanged}\;\mathrm{compound}\;\mathrm{concentration}\;\mathrm{in}\;\mathrm{microsome}}$	(47)		
${\mathrm{CL}}_{\mathrm{int},in\;vivo,\mathrm{pred}}={\mathrm{CL}}_{\mathrm{int},in\;vitro}\cdot \mathrm{MPR}=\frac{V_{\max}}{K_{M}}\cdot \mathrm{MPR}$	(48)	Microsomal protein recovery (MPR) ratio was incorporated in IVIVE R_B/P_ refers to blood to plasma ratio	[67]
$\mathrm{MPR}\;\left(\mathrm{mg}\;\mathrm{protein}/g\;\mathrm{liver}\right)=\frac{\mathrm{Liver}\;\mathrm{homogenate}\;\mathrm{CYP}\;\mathrm{content}\;\left(\mathrm{nmol}/g\;\mathrm{liver}\right)}{\mathrm{Microsomal}\;\mathrm{CYP}\;\mathrm{content}\;\left(\mathrm{nmol}/\mathrm{mg}\;\mathrm{protein}\right)}$	(49)		
${\mathrm{CL}}_{\mathrm{int}.in\;vivo,\mathrm{obs}}=\frac{\mathrm{CL}}{f_{p}\cdot R_{B/P}}$	(50)		
$P450\;\mathrm{content}\;\mathrm{correcting}\;\mathrm{factor}=\frac{P450\;\mathrm{isozyme}\;\mathrm{content}/g\;\mathrm{liver}}{P450\;\mathrm{isozyme}\;\mathrm{content}/\mathrm{mg}\;\mathrm{protein}}$	(51)	CYP abundance was incorporated in IVIVE	[68]
$\mathrm{RAF}=\frac{V_{\max}\left(\mathrm{HML}\right)}{V_{\max}\left(\mathrm{rhCYP}\right)}$	(52)	Relative activity factor (RAF) introduced for scaling rhCYP data to HLM Modified RAF taking into account of K_m_	[69,70]
$\mathrm{RAF}=\frac{{\mathrm{CL}}_{\mathrm{int}}\left(\mathrm{HML}\right)}{{\mathrm{CL}}_{\mathrm{int}}\left(\mathrm{rhCYP}\right)}$	(53)		
${\mathrm{CL}}_{\mathrm{int}}=[\overset{n}{\underset{j=1}{\sum }}\left(\overset{n}{\underset{i=1}{\sum }}\frac{V_{\max}{\left({\mathrm{rhCYP}}_{j}\right)}_{i}\times {\mathrm{RAF}}_{\mathrm{ij}}\left(V_{\max}\right)}{K_{m}{\left({\mathrm{rhCYP}}_{j}\right)}_{i}}\right)]\times \mathrm{MPPGL}\times \mathrm{Liver}\;\mathrm{weight}$	(54)		
$\mathrm{ISEF}=\frac{V_{\max_{\mathrm{ji}}}\left(\mathrm{HML}\right)}{V_{\max_{i}}\left({\mathrm{rhCYP}}_{j}\right)\times {\mathrm{CYP}}_{j}\mathrm{abundance}\;\left(\mathrm{HLM}\right)}$	(55)	Inter-system extrapolation factor (ISEF) is introduced for scaling rhCYP data to HLM	[69]
${\mathrm{CL}}_{\mathrm{int}}=[\overset{n}{\underset{j=1}{\sum }}\left(\overset{n}{\underset{i-1}{\sum }}\frac{V_{\max_{i}}\left({\mathrm{rhCYP}}_{j}\right)\times {\mathrm{CYP}}_{j}\;\mathrm{abundance}}{K_{m}{\left({\mathrm{rhCYP}}_{j}\right)}_{i}}\right)]\times \mathrm{MPPGL}\times \mathrm{Liver}\;\mathrm{weight}$	(56)		
${\mathrm{CL}}_{H}=\frac{R_{B/P}\cdot Q_{H}\cdot {\mathrm{CL}}_{\mathrm{int},\mathrm{liver},\mathrm{human}}\cdot f_{p}\cdot F_{I}}{R_{B/P}\cdot Q_{H}+{\mathrm{CL}}_{\mathrm{int},\mathrm{liver},\mathrm{human}}\cdot f_{p}\cdot F_{I}}$	(57)	The ionization factor is incorporated into the IVIVE F_I_ is an ionization factor Subscript letter IW denotes intracellular water Upper letter i and n indicate compounds of ionized and neutral forms, respectively	[71]
$F_{I}=\frac{f_{p}^{n}}{f_{\mathrm{IW}}^{n}}=\frac{1-f_{p}^{i}}{1-f_{\mathrm{IW}}^{i}}$	(58)		
$f_{\mathrm{acid}}^{i}=\frac{\left[A^{-}\right]}{{\left[\mathrm{AH}\right]}_{0}}=\frac{1}{1+10^{pK_{a}-\mathrm{pH}}}$	(59)		
$f_{\mathrm{base}}^{i}=\frac{\left[{\mathrm{BH}}^{+}\right]}{{\left[B\right]}_{0}}=\frac{1}{1+10^{\mathrm{pH}-pK_{a}}}$	(60)		
${\mathrm{CL}}_{H}=\frac{Q_{H}\cdot {\mathrm{CL}}_{\mathrm{int},\mathrm{liver},\mathrm{human}}\cdot f_{u,\mathrm{liver}}/f_{u,\mathrm{mic}}}{Q_{H}+{\mathrm{CL}}_{\mathrm{int},\mathrm{liver},\mathrm{human}}\cdot f_{u,\mathrm{liver}}/f_{u,\mathrm{mic}}}$	(61)	The unbound fraction into the liver (f_u,liver_) is incorporated into the IVIVE Plasma to whole liver concentration ratio (PLR) = 13.3	[72]
$f_{u,\mathrm{liver}}=\frac{\mathrm{PLR}\cdot f_{u,p,\mathrm{app}}}{1+\left(\mathrm{PLR}-1\right)\cdot f_{u,p,\mathrm{app}}}$	(62)		
${\mathrm{CL}}_{\mathrm{int},\mathrm{liver},invitro}={\mathrm{PS}}_{\mathrm{uptake},\mathrm{total}}\cdot \frac{{\mathrm{CL}}_{\mathrm{met}}+{\mathrm{PS}}_{\mathrm{bile}}}{{\mathrm{CL}}_{\mathrm{met}}+{\mathrm{PS}}_{\mathrm{efflux},\mathrm{total}}+{\mathrm{PS}}_{\mathrm{bile}}}$	(63)	Physiologically-based IVIVE model Total apparent uptake clearance (PS_uptake,total_) consists of saturable and/or non-saturable processes CL_met_ and PS_bile_ refer to metabolic and biliary clearance, respectively Apparent sinusoidal total efflux clearance from the intracellular side of hepatocytes back into blood (PS_efflux, total_) consists of saturable and/or non-saturable processes	[73]
${\mathrm{CL}}_{H}=\frac{Q_{H}\cdot {\mathrm{CL}}_{\mathrm{int},\mathrm{liver},invitro}\cdot f_{p}}{Q_{H}+{\mathrm{CL}}_{\mathrm{int},\mathrm{liver},in\;vitro}\cdot f_{p}}$	(64)		
${\mathrm{fn}}_{H}={\mathrm{fn}}_{\sec}+{\mathrm{fn}}_{\mathrm{met}}$	(65)	Provide the method for the prediction of total clearance and relative elimination contributions The fn_H,_ fn_sec,_ and fn_met_ refers to a fractional contribution of hepatic, biliary, and metabolic elimination to overall clearance PS_inf, act_ and PS_inf, pas_ refer to the sinusoidal active and passive influx clearance, respectively Sinusoidal efflux from hepatocytes back into blood (PS_eff_) is assumed to occur via passive diffusion, therefore PS_eff_ = PS_inf,pas_ CL_int,sec_ and CL_int,met_ refer to intrinsic secretory and metabolic clearance, respectively PS_inf_ equals to the sum of PS_inf,act_ and PS_inf,pas_ which are determined by suspension of pooled human hepatocytes (unit: mL/min/kg)	[8]
${\mathrm{fn}}_{H}=1-e^{-0.01741{\mathrm{PS}}_{\inf}}$	(66)		
${\mathrm{fn}}_{\mathrm{met}}=1-e^{-0.01521{\mathrm{PS}}_{\inf,\mathrm{pas}}}$	(67)		
${\mathrm{CL}}_{\mathrm{renal}}={\mathrm{CL}}_{\mathrm{total}}-{\mathrm{CL}}_{H}$	(68)		
${\mathrm{CL}}_{\mathrm{total}}=\frac{{\mathrm{CL}}_{H}}{{\mathrm{fn}}_{H}}$	(69)		
${\mathrm{CL}}_{\mathrm{int},in\;vitro}=\frac{\left({\mathrm{PS}}_{\inf,\mathrm{act}}+{\mathrm{PS}}_{\inf,\mathrm{pas}}\right)\cdot \left({\mathrm{CL}}_{\mathrm{int},\sec}+{\mathrm{CL}}_{\mathrm{int},\mathrm{met}}\right)}{{\mathrm{PS}}_{\mathrm{eff},\mathrm{total}}+{\mathrm{CL}}_{\mathrm{int},\sec}+{\mathrm{CL}}_{\mathrm{int},\mathrm{met}}}$	(70)		
${\mathrm{CL}}_{H}=\frac{Q_{H}\cdot {\mathrm{CL}}_{\mathrm{int},in\;vitro}\cdot f_{p}}{Q_{H}+{\mathrm{CL}}_{\mathrm{int},in\;vitro}\cdot f_{p}}$	(71)		

* Each comment corresponds to all the equations within each major section of the table defined by horizontal lines.

**Table 3:** Methods for prediction of clearance (CL) using allometric scaling (AS).

Method	Equation		Comments *	Ref.
Simple AS	$\mathrm{CL}=a{\left(W\right)}^{b}$	(74)	Select a proper equation by the rule of exponent (ROE) W and BW represent body and brain weight, respectively	-
AS with MLP ^1^	$\mathrm{CL}\cdot \mathrm{MLP}=a{\left(W\right)}^{b}$	(75)		-
AS with BW	$\mathrm{CL}\cdot \mathrm{BW}=a{\left(W\right)}^{b}$	(76)		[89]
Rule of exponent	If the exponent is 0.55 to 0.7, then use the simple AS, Equation (74)			[90]
	If the exponent is 0.71 to 1, then use the MLP, Equation (75)			
	If the exponent is more than 1, then use the BW, Equation (76)			
Two-term method	$\mathrm{CL}=\theta {\left(W\right)}^{a}\cdot {\left(\mathrm{BW}\right)}^{b}$	(77)	θ is a constant, which is determined by multiple regression analysis	[91]
Multiexponential	${\mathrm{CL}}_{\mathrm{human}}={\mathrm{aW}}^{b}+\left[\left(\frac{1-\frac{3}{2}b}{1-\frac{1}{2}b}\right)\right]{\mathrm{aW}}^{0.9}$	(78)	The unit of CL is mL/min	[92]
Normalized AS	${\mathrm{CL}}_{\mathrm{animal}}\frac{{\mathrm{CL}}_{\mathrm{int},\mathrm{human}}}{{\mathrm{CL}}_{\mathrm{int},\mathrm{animal}}}=a{\left(W\right)}^{b}$	(79)	CL_int_ refers the unbound CL_int_ in microsomes or hepatocytes in species and humans	[93]
One species AS	${\mathrm{CL}}_{\mathrm{human}}={\mathrm{CL}}_{\mathrm{animal}}\cdot {\left(\frac{W_{\mathrm{human}}}{W_{\mathrm{animal}}}\right)}^{b}$	(80)	The exponent b is a constant 0.75, which is physiologically relevant value (e.g., blood flow, filtration, etc.)	[94,95]
One species AS	${\mathrm{CL}}_{\mathrm{pred}}=0.152\cdot {\mathrm{CL}}_{\mathrm{rat}}\cdot \left(\frac{W_{\mathrm{human}}}{W_{\mathrm{rat}}}\right)$	(81)	Predict the CL of bound drug	[90]
	${\mathrm{CL}}_{\mathrm{pred}}=0.41\cdot {\mathrm{CL}}_{\mathrm{dog}}\cdot \left(\frac{W_{\mathrm{human}}}{W_{\mathrm{dog}}}\right)$	(82)		
	${\mathrm{CL}}_{\mathrm{pred}}=0.407\cdot {\mathrm{CL}}_{\mathrm{monkey}}\cdot \left(\frac{W_{\mathrm{human}}}{W_{\mathrm{monkey}}}\right)$	(83)		
Two species AS	${\mathrm{CL}}_{\mathrm{pred}}=a_{\mathrm{rat}-\mathrm{dog}}\cdot W_{\mathrm{human}}{}^{0.628}$	(84)	Predict the CL of bound drug	
	${\mathrm{CL}}_{\mathrm{pred}}=a_{\mathrm{rat}-\mathrm{monkey}}\cdot W_{\mathrm{human}}{}^{0.650}$	(85)		
Hepatic liver method	${\mathrm{CL}}_{\mathrm{pred}}={\mathrm{CL}}_{\mathrm{animal}}\cdot \left(\frac{Q_{H,\mathrm{human}}}{Q_{H,\mathrm{animal}}}\right)$	(86)		[96]
FCIM ^2^	$\mathrm{CL}=33.35\times {\left(\frac{a}{{\mathrm{Rf}}_{u}}\right)}^{0.77}$	(87)	Rf_u_ is the f_u_ ratio between rats and humans and a is the coefficient form AS The unit of CL is mL/min	[97]
QSAR ^3^	$\begin{array}{ll} {\mathrm{LogCL}}_{\mathrm{pred}} & =0.433\cdot \log({\mathrm{CL}}_{\mathrm{rat}})\\ & +1.0\cdot \log({\mathrm{CL}}_{\mathrm{dog}})\\ & -0.00627\cdot \mathrm{MW}+0.189\cdot \mathrm{Ha}\\ & -0.00111\cdot \log({\mathrm{CL}}_{\mathrm{dog}})\cdot \mathrm{MW}\\ & +0.0000144\cdot {\mathrm{MW}}^{2}\\ & -0.0004\cdot \mathrm{MW}\cdot \mathrm{Ha}-0.707 \end{array}$	(88)	The unit of observed and predicted CL value is mL/min/kg	[98]
	$\begin{array}{l} {\mathrm{LogCL}}_{\mathrm{po},\mathrm{pred}}\\ =-0.5927+0.7386\log\left({\mathrm{CL}}_{\mathrm{po},\mathrm{rat}}\right)\\ +0.5040\log\left({\mathrm{CL}}_{\mathrm{po},\mathrm{dog}}\right)\\ +0.06014\mathrm{clogP}\\ -0.1862\log\left({\mathrm{CL}}_{\mathrm{po},\mathrm{dog}}\right)\times \mathrm{clogP}\\ +0.02893\mathrm{MW}\times \mathrm{clogP}\\ +0.02893\mathrm{MW}\times \mathrm{clogP}\\ +0.02551\log\left({\mathrm{CL}}_{\mathrm{po},\mathrm{rat}}\right)\\ \times \log\left({\mathrm{CL}}_{\mathrm{po},\mathrm{rat}}\right)\mathrm{clogP}\\ -0.03029\log\left({\mathrm{CL}}_{\mathrm{po},\mathrm{rat}}\right)\times \log\left({\mathrm{CL}}_{\mathrm{po},\mathrm{dog}}\right)\\ \times \mathrm{Ha}\\ -0.03051\log\left({\mathrm{CL}}_{\mathrm{po},\mathrm{rat}}\right)\times \mathrm{MW}\times \mathrm{clogP}\\ +0.08461\log\left({\mathrm{CL}}_{\mathrm{po},\mathrm{dog}}\right)\times \log\left({\mathrm{CL}}_{\mathrm{po},\mathrm{dog}}\right)\\ \times \log\left({\mathrm{CL}}_{\mathrm{po},\mathrm{dog}}\right)\\ -0.2510\log\left({\mathrm{CL}}_{\mathrm{po},\mathrm{dog}}\right)\times \log({\mathrm{CL}}_{\mathrm{po},\mathrm{dog}})\\ \times \mathrm{MW}\\ +0.06061\log\left({\mathrm{CL}}_{\mathrm{po},\mathrm{dog}}\right)\times \log\left({\mathrm{CL}}_{\mathrm{po},\mathrm{dog}}\right)\\ \times \mathrm{Ha}\\ +0.04607\log\left({\mathrm{CL}}_{\mathrm{po},\mathrm{dog}}\right)\times \mathrm{clogP}\times \mathrm{clogP}\\ -0.003596\mathrm{clogP}\times \mathrm{clogP}\times \mathrm{Ha}\\ +0.0005963\mathrm{clogP}\times \mathrm{Ha}\times \mathrm{Ha} \end{array}$	(89)	The unit of observed and predicted oral CL value is mL/min/kg	[99]

* Each comment corresponds to all the equations within each major section of the table defined by horizontal lines. ^1^ The maximum life-span potential (MLP) is calculated by the equation MPL (years) = 185.4BW^0.636^W^−0.225^ [100]. ^2^ Fraction unbound intercept correction method. ^3^ Quantitative structure activity relationship (QSAR) consist of physicochemical properties, such as molecular weight (MW), partition coefficient (cLogP), and number of hydrogen-bound acceptors (Ha).

**Table 4:** Methods for prediction of volume of distribution (V_d_).

Method	Equation		Comment *	Ref.
Simple AS	$V=a{\left(W\right)}^{b}$	(93)	The prediction of V_d_ is well predicted equally with using two species in AS	[108]
Average fraction unbound in tissue ^1^	$V=V_{\mathrm{Plasma}}\left(1+R_{E/I}\right)+f_{u}\cdot V_{P}\left(\frac{V_{E}}{V_{p}}-\frac{V_{R}\cdot f_{u}}{\alpha_{R}}\right)$	(94)	It is useful to analyze and predict an alteration in apparent V_d_ then identify the cause of alteration. It is particularly useful for drugs with low V_d_ (<15 L or 0.2 L/kg)	[119]
Proportionality	$V_{\mathrm{human},\;\mathrm{pred}}=\frac{V_{\mathrm{animal}}\cdot f_{u,\;\mathrm{human}}}{f_{u,\;\mathrm{animal}}}$	(95)	It is assumed that the volume of distribution at a steady state of free drug is identical between species	[120]
One species AS	$V_{\mathrm{human},\;\mathrm{pred}}=-0.35V_{\mathrm{rat}}{}^{0.91}$	(96)	Statistical modeling is applied in this model	[121]
QSAR	$\begin{array}{ll} \log\left({\mathrm{Vd}}_{\mathrm{ss},\mathrm{human}}\right) & =0.1859\cdot \log\left({\mathrm{Vd}}_{\mathrm{ss},\;\mathrm{rat}}\right)\times \log\left({\mathrm{Vd}}_{\mathrm{ss},\;\mathrm{rat}}\right)\\ & -0.3887\cdot \log\left({\mathrm{Vd}}_{\mathrm{ss},\;\mathrm{rat}}\right)\times \log\left(\mathrm{MW}\right)\\ & +0.3089\cdot \log\left({\mathrm{Vd}}_{\mathrm{ss},\mathrm{dog}}\right)\times \log\left(\mathrm{MW}\right)\\ & +0.003306\cdot \log\left(\mathrm{MW}\right)\times c\log P\\ & +1.71 \end{array}$	(97)	Vd_ss, human_ (mL/kg) is predicted by QSAR modeling with quadratic term descriptors	[122]

* Each comment corresponds to all the equations within each major section of the table defined by horizontal lines. ^1^ Where V_d_ is apparent volume of distribution, V_plasma_ is plasma volume, V_E_ is extracellular space minus the plasma, V_R_ is physical volume into which the drug distributes minus the extracellular space, f_u_ is the fraction unbound in plasma, and R_E/I_ is the ratio of distributed albumin in the extravascular space to that in the intravascular space. It is 1.4. α_R_ equals to C_u_/C_R_ where C_u_ is unbound drug concentration at distribution equilibrium and C_R_ is concentration in V_R_.

**Table 5:** Methods for prediction of absorption parameters.

Method	Equation		Comments *	Ref.
AS	$k_{a}={\mathrm{animal}\;k}_{a}\times {\left(\frac{W_{\mathrm{human}}}{W_{\mathrm{animal}}}\right)}^{-0.25}$	(102)	The unit of k_a_ is h in time^−1^	[88]
QSAR^1^	${\mathrm{logP}}_{\mathrm{eff}}=-2.883-0.01\mathrm{PSA}+0.192{\mathrm{logD}}_{5.5}-0.239\mathrm{HBD}$	(103)	The choice of model for prediction depends on the availability of descriptor data Effective permeability in 10^−4^ cm/s	[124]
	${\mathrm{logP}}_{\mathrm{eff}}=-2.546-0.011\mathrm{PSA}-0.278\mathrm{HBD}$	(104)		
	${\mathrm{logP}}_{\mathrm{eff}}=-3.067+0.162\mathrm{clogP}-0.01\mathrm{PSA}-0.235\mathrm{HBD}$	(105)		
Use of Caco2 data ^2^	$P_{\mathrm{eff},\mathrm{human}}=0.4926\log P_{\mathrm{eff},\mathrm{Caco}2}-0.1454\;\left(\mathrm{at}\;\mathrm{pH}=7.4\right)$	(106)	All tested drugs	[125]
	$P_{\mathrm{eff},\mathrm{human}}=0.6532\log P_{\mathrm{eff},\mathrm{Caco}2}-0.3036\;\left(\mathrm{at}\;\mathrm{pH}=6.5\right)$	(107)		
	$P_{\mathrm{eff},\mathrm{human}}=0.6836\log P_{\mathrm{eff},\mathrm{Caco}2}-0.5579\;\left(\mathrm{at}\;\mathrm{pH}=7.4\right)$	(108)	Only passively diffused drugs	
	$P_{\mathrm{eff},\mathrm{human}}=0.7254\log P_{\mathrm{eff},\mathrm{Caco}2}-0.5441\;\left(\mathrm{at}\;\mathrm{pH}=6.5\right)$	(109)		
	$P_{\mathrm{eff},\mathrm{human}}=0.4898\log P_{\mathrm{eff},\mathrm{Caco}2}+0.3311\;\left(\mathrm{at}\;\mathrm{pH}=7.4\right)$	(110)	Only carrier-mediated drugs	
	$P_{\mathrm{eff},\mathrm{human}}=0.542\log P_{\mathrm{eff},\mathrm{Caco}2}+0.06\;\left(\mathrm{at}\;\mathrm{pH}=6.5\right)$	(111)		
Sinko et al. ^5^	$k_{a}=\frac{2P_{\mathrm{eff}}}{R}$	(112)	The absorption rate constant is proportional to the P_eff_	[126]
Mechanism based modeling ^3^	$F_{a,\mathrm{pred}}=0.884F_{a,\exp}+7.47$	(113)	F_a_ is expressed as percent unit The equation is the result of the correlation between F_a,pred_ and F_a,exp_	[127]
	$k_{a,\mathrm{eq}}=\frac{P_{m}S}{V_{c}}$	(114)	k_a,eq_ is expressed as the unit of min^−1^ k_a,eq_ is a key determinant for F_a_ and can be used as PK modeling	
	$F_{a}=\frac{k_{a,\mathrm{eq}}}{k_{i}+k_{a,\mathrm{eq}}}$	(115)		
Compartmental absorption and transit model ^4^	$F_{a}=1-{\left(1+0.54P_{\mathrm{eff}}\right)}^{-7}$	(116)	F_a_ is expressed as the fractional value.	[128]

* Each comment corresponds to all the equations within each major section of the table defined by horizontal lines. ^1^ In this equation, passive intestinal absorption in humans was predicted. Abbreviations are: P_eff_, effective permeability; PSA, polar surface area; logD_5.5_, octanol/water distribution coefficient at pH 5.5; HBD, number of hydrogen bond donors; clogP, calculated logP value. ^2, 5^ P_eff_ is calculated by the equation of P_eff_ = Q(1-C_out_/C_in_)/2πRL, where P_eff_ is effective permeability, Q is perfusion rate (mL/min), C_out_ and C_in_ are outlet and inlet drug concentration, respectively, R is the radius of human jejunum (1.75 cm) [129], and L is the length of perfusion segment (10 cm). Caco2 permeability and human effective permeability are expressed with values of ×10^−6^ cm/s and ×10^−4^ cm/s, respectively. ^3^ k_a,eq_ is the equilibrium solution for k_a_, P_m_ is drug permeability across intestinal mucosa (×10^−6^ cm/s), S is the absorptive surface area which is set at 200 m^2^, V_c_ is the volume of distribution in well-perfused organs, k_i_ is the rate constant of intestinal transit, which is set to be 5.025 × 10^−3^ min^−1^ as an inverse value of the average transit time [130] in human small intestine (approximately 199 min). ^4, 5^ P_eff_ is human effective permeability in cm/h.

## Abbreviations

ADME	absorption, distribution, metabolism, excretion
AS	allometric scaling
AUC_0-inf_	area under the concentration-time curve from zero to infinity
BDDCS	biopharmaceutics drug disposition classification system
BW	brain weight
CB	drug concentration in blood
CL	clearance
CLH	hepatic clearance
CL_int_	intrinsic clearance
CL_int, H_	hepatic intrinsic clearance
CL_int,H, human_	human hepatic intrinsic clearance
CL_int,H, pred_	predicted hepatic intrinsic clearance
CL_int,in vitro_	in vitro intrinsic clearance
CL_int,met_	intrinsic metabolic clearance
CL_int,sec_	intrinsic secretory clearance
CL_met_	metabolic clearance
cLogP	partition coefficient
clogP	calculated logP
CL_org_	organ clearance
C_P_	drug concentration in plasma
C_R_	concentration in VR
C_RBC_	drug concentration in red blood cells
C_u_	unbound drug concentration at distribution equilibrium
DMPK	drug metabolism and pharmacokinetic
D_N_	dispersion number
ECM	extended clearance model
ER	extraction ratio
ER_H_	hepatic extraction ratio
ER_H, obs_	observed hepatic extraction ratio
ER_H, pred_	predicted hepatic extraction ratio
F	fraction of absorption
$f_{\mathrm{IW}}^{i}$	unbound fraction in the intracellular water of ionized compound
$f_{\mathrm{IW}}^{n}$	unbound fraction in the intracellular water of neutral compound
$f_{p}^{i}$	unbound fraction in the plasma of ionized compound
$f_{p}^{n}$	unbound fraction in the plasma of neutral compound
F_a_	fraction of absorption
f_b_	unbound fraction in blood
FCIM	fraction unbound intercept correction method
F_H_	hepatic availability
F_I_	ionization factor
f_nH_	fractional contribution of hepatic elimination
f_nmet_	fractional contribution of metabolic elimination
f_nsec_	fractional contribution of biliary elimination
f_p_	unbound fraction in plasma
F_PO_	oral bioavailability
f_u, mic_	non-specific binding factor to microsomes
f_u,liver_	unbound fraction into the liver
Ha	number of hydrogen-bond acceptors
HBD	number of hydrogen-bond donor
Hct	hematocrit
HLM	human liver microsomes
IIV	inter-individual variability
ISEF	inter-system extrapolation factor
ISTD	internal standard
IVIVE	in vitro-in vivo extrapolation
IW	intracellular water
k_a_	absorption rate constant
k_a,eq_	equilibrium solution for k_a_
k_i_	the rate constant of intestinal transit
K_m_	Michaelis constant
L	length of perfusion segment
MA	multiexponential allometric scaling
MLBF	monkey liver blood flow
MLP	maximum life-span potential
MPPGL	microsomal protein per gram of liver
MPR	microsomal protein recovery
MRT	mean residence time
MW	molecular weight
NCA	non-compartmental analysis
P_eff_	effective permeability
PK	pharmacokinetic
PLR	plasma to whole liver concentration ratio
P_m_	drug permeability across intestinal mucosa
PSA	polar surface area
PS_bile_	biliary clearance
PS_efflux, total_	apparent sinusoidal total efflux clearance from the intracellular side of hepatocytes back into blood
PS_inf, act_	sinusoidal efflux from hepatocytes back into blood
PS_uptake,total_	total apparent uptake clearance
Q_H_	hepatic liver flow
QSAR	quantitative structure activity relationship
R	radius of human jejunum
RAF	relative activity factor
R_B/P_	blood to plasma ratio
R_E/I_	ratio of distributed albumin in the extravascular space to that in the intravascular space
R_fu_	unbound fraction in plasma ratio between rats and humans
rhCYP	recombinant human CYP system
R_N_	efficiency number
ROE	rule of exponent
S	absorptive surface area
t_1/2_	half-life
V	volume of distribution
V_area_, V_β_	volume of distribution by area
V_c_	volume of distribution of central compartment
V_E_	extracellular space volume minus the plasma volume
V_max_	maximal rate of the reaction
V_met_	metabolic rate
V_plasma_	plasma volume
V_R_	physical volume into which the drug distributes minus the extracellular space
V_ss_	volume of distribution at steady state
W	body weight
ε^2^	variance for each sinusoid in the whole liver
